# Presence of *Trichomonas spp.* in oral ulcerations of a patient with kidney transplant. A case report

**DOI:** 10.4317/jced.56922

**Published:** 2020-12-01

**Authors:** Ganna Dmytrenko, Lorena Tana, María-Victoria Cachau, Martín Bravo, Silvina Gonzalez, Fernando Correa, Javier Fernandez-Solari, Andrea De Laurentiis

**Affiliations:** 1 Centre of Pharmacological and Botanical Studies, CEFYBO-CONICET-UBA, School of Medicine, University of Buenos Aires, Argentina; 2 Physiology Dept., School of Dentistry, University of Buenos Aires, Argentina; 3 National Scientific and Technical Research Council (CONICET), Argentina; 4 High Complexity Transplant Institute, Nephrology Argentina; 5 Pathology Dept., José de San Martín Clinical Hospital, Buenos Aires, Argentina; 6 Stomatology Dept., José de San Martín Clinical Hospital, Buenos Aires, Argentina

## Abstract

Mucosal ulcerations are an oral complication that can often affect kidney transplant patients, mostly due to the effect of immunosuppression. It has been frequently reported drug-induced ulceration or lymphoproliferative disorders with buccal manifestations however, some unusual disorders should also be considered, such as fungal infections, viruses, as well as opportunistic infection by other microorganisms. Determining the etiology and differential diagnose from other causes of mouth ulcers is very important for the adequate treatment of said lesion. Dental health of patients should also be taken into the account prior to the transplant surgery, since periodontal pockets are the main niche of microbial reservoir. Moreover, mixed with oral microbiota, parasites such as *Trichomonas spp.* can be found in the dental plaque of patients with periodontal disease. Particularly, *Trichomonas spp.* are anaerobic motile-flagellated protozoa that can both induce tissue damage and exacerbate preexistent injuries in vaginal and oral mucosa. Parasitic infection in the oral cavity has not been well studied and it is thought to be underreported. In the present study we report the first case in literature of presence of *Trichomonas spp.* as a potential etiological factor of the oral ulcerations of a kidney transplanted patient that remitted after antibiotic treatment.

** Key words:**Immunosuppression, protozoan, buccal lesion, oral mucosa, kidney transplant.

## Introduction

Mucosal ulcerations are an oral complication that can often affect kidney transplant patients and the adequate treatment depends on the correct determination of its etiology. Differential diagnosis of lesions in immunocompromised patients covers some unusual disorders such as drug-induced ulcers, lymphoproliferative disorders, fungal infections, viruses, as well as opportunistic infection by other microorganisms that could normally constitute saprophyte microbiota (1).

Among oral microbiota, *Trichomonas tenax*, an anaerobic motile-flagellated protozoan is a parasite classified in the same genus as *Trichomonas vaginalis* that can be found in dental plaques. In humans, *Trichomonas vaginalis* causes disease in the genitals and can both induce tissue damage and benefit from a modified micro-environment or immunosuppression to grow and exacerbate preexistent injuries (2). Regarding *Trichomonas tenax* pathogenicity, it could be hypothesized that it acts by similar mechanisms than *Trichomonas vaginalis* since it could be a variant of that parasite that developed genotypic changes and acquired a new phenotype, suitable to the oral cavity (3). Additionally, several strains of *Trichomonas tenax* isolated from periodontal pockets expressed several proteinases that could disrupt the host tissue integrity and induce cell lysis, promoting oral tissue destruction. Yamamoto *et al.* demonstrated that *Trichomonas tenax* secretes various proteinases with characteristics of cysteine-proteinases which can attack parts of the mammalian cells causing membrane blebbing and apoptotic bodies (4). Ribeiro *et al.* confirmed the ability of the flagella to adhere to periodontal epithelial cells and cause damage and cell apoptosis. *Trichomonas vaginalis* has also been found in the oral cavity, but failed to elicit damage on epithelial and fibroblast gum cells *in vitro* (5).

The most frequently used method for the identification of these parasites is direct microscopy. However, it is insufficient to discriminate parasite species since they are morphologically close. In order to precisely identify parasite species, Polymerase Chain Reaction (PCR) and sequencing of its products are needed. Nevertheless, microscopic observation method is used at chairside for diagnosis and treatment management (2). Here we report one case of *Trichomonas spp.* presence in the oral ulcerations of a kidney transplanted patient.

## Material and Methods

A 48-year-old man underwent a renal transplant. The patient had chronic renal failure secondary to polycystic kidney disease and underwent hemodialysis during two years prior to transplantation. Prior to surgery, other medicals findings were mild chronic mitral regurgitation, left ventricular hypertrophy, erythematous gastroduodenopathy with benign antral polyp and hyperparathyroidism secondary to kidney disease.

The organ was obtained from a cadaveric cytomegalovirus (CMV)-seropositive donor. After transplantation the patient was placed on long-term immunosuppressive therapy. His complete current medication is cited in Table 1. He had no post-transplant complications and showed no evidence of opportunistic infection.

**Table 1:**
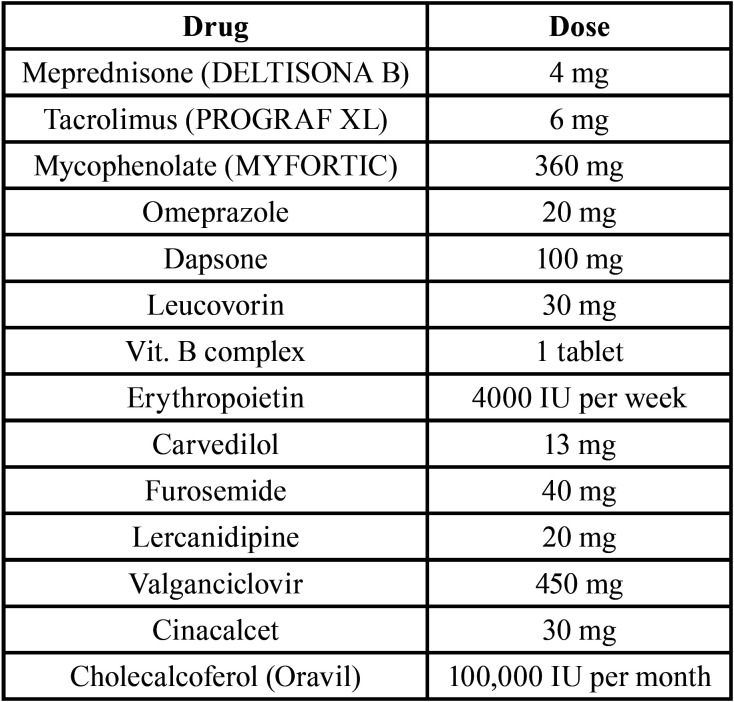
Current medication at the moment of oral examination expressed as daily dose.

## Results

-Clinical presentation: Four months after transplantation, the patient was referred by his attending physician due to ulcerative lesions on the left labial and anterior jugal mucosa with 10 days of evolution and mild pain. His post-transplant course had been relatively uneventful until the appearance of these lesions. His last lab test showed mild leukopenia (2800 cells/mm3). All other parameters were within normal range.

**Figure 1:**
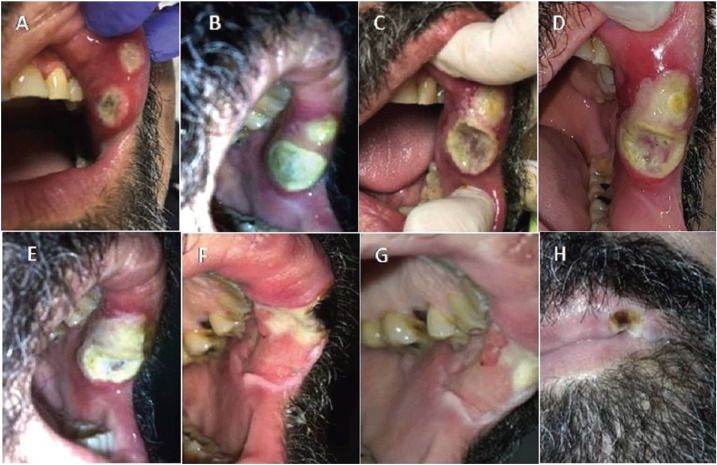
Evolution of the oral ulcerations on the jugal mucosa. A) Clinical aspect on the first stomatological consultation. B) 48 h of evolution showing increased size and almost confluent lesions. C) 10 days of evolution. Detached necrotic layer has been removed. D) Oral lesions aspect when systemic metronidazole treatment was started. E-H) Healing process with marked improvement of clinical aspect of lesions.

The intraoral examination revealed two lesions with large diameter of 1 cm and 1.5 cm respectively, circumscribed with elevated regular edges and slightly erythematous surrounding mucosa, soft on palpation. The center had a necrotic appearance with a yellowish color and dark brown pigmentation in the largest one (Fig. 1A). Fever, systemic symptoms and regional lymph nodes were not detected.

During the anamnesis, it was revealed that the patient comes from a rural area where he worked as a chicken farm consultant and owned a pig and a peacock farm.

-Differential diagnosis: Given the clinical appearance of the lesions and the patient’s medical history, the following options were considered in the presumptive diagnosis: drugs or mycosis-induced ulcers, CMV or Epstein Bar Virus (EBV) lesions, post-transplant lymphoproliferative disorder (PTLD).

Periodontal prophylaxis was performed to remove the bacterial load of the oral cavity to avoid worsening of the lesions. Further indication was local miconazole 2% four times/day and mouthwash with chlorhexidine 20% twice a day. An interconsultation was performed with the attending physician who augmented dosage of valganciclovir and requested viral load for CMV, EBV, herpes simplex virus (HSV) and varicella-zoster virus (VZV). Forty-eight hours after the first stomatological appointment, no improvement of the lesions was obtained. Conversely, an increase in the size of both lesions was observed with more edematous surrounding tissue (Fig. 1B). The patient complained about the appearance of a fetid smell from the lesions. A superinfection with anaerobic bacteria was suspected and topical treatment with metronidazole 1% was added. Eight days after the first appointment, the necrotic layer turned to a greenish color and was detached from the lesion. PCR analysis reports for HSV, VZV and CMV resulted negative; therefore, the dosage of valganciclovir was reduced to prophylaxis as previously.

Ten days after the dentist first appointment, the pain increased but the fetid smell completely disappeared. The lesions did not decrease in size but they became more penetrating (Fig. 1C). Microbiological analysis and biopsy were requested suspecting opportunistic infections due to mild leukopenic condition. Thorax tomography was performed to discard a lung fungal primary infection.

The patient’s leukopenic condition was handled withdrawing mycophenolate and adding 3 doses of filgastrim (300 ug) to stimulate white blood cells. After ten days, the white blood cells count arose to 5000 cells/mm3 and mycophenolate was reinstated to habitual dose.

**Figure 2:**
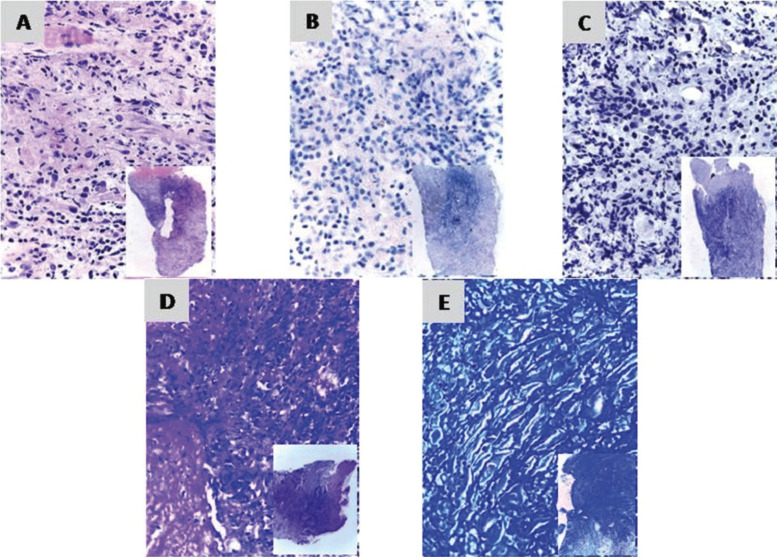
Photomicrograph of the oral biopsy. A) non-specific ulcer with lymphoplasmacytic infiltration with diffused neutrophils and eosinophils (H&E). B) Immunostaining for CMV1 and C) for CMV2 without positive marks. D) Ziehl-Neelsen stain and E) Giemsa stain. Magnification 400x.

The biopsy showed non-specific ulcer with a fibrinoleukocyte layer, lymphoplasmacytic infiltration with diffused neutrophils and eosinophils. Ziehl Neelsen, Giemsa and immunostaining for CMV did not show positive results for any specific microorganisms (Fig. 2). A microbiologic analysis showed the presence of Streptococcus viridians and normal oral microbiota. However, in the direct microscopic observation of moist sample, a motile pyriform protozoon with flagellar movements compatible with *Trichomonas spp.* was observed (Supplemental video available at (URL/link)). Consequently, an infection with *Trichomonas spp.* was considered and treatment with metronidazole 250 mg orally 3 times a day for 7 consecutive days was started. The lesions began to notably remit (Fig. 1 D-H).

## Discussion

Polymedication and induced immunosuppression after kidney transplant predispose to a greater susceptibility to infections of bacterial, fungal and viral origin. Since the transplanted organ was obtained from a CMV seropositive donor, a viral etiology for the ulcers was considered first. The ulcerative CMV lesions in immunosuppressed patients were reported in different anatomical sites, including oral cavity (6). In our patient, this opportunistic infection was discarded as etiological factor of the oral lesions after repeated negative results for CMV detection in serum and biopsy samples.

While waiting for the lab results concerning the CMV detection, we considered a superinfection with this virus and an opportunistic fungal pathogen. A thorax tomography was performed to detect possible foci of pulmonary mycosis and resulted negative. *Candida* sp. is found as part of oral microbiota and can cause lesions as an opportunistic agent in immunosuppressed patients. It must be kept in mind at the time of a differential diagnosis of oral ulcers of unknown origin (7). We did not observed hyphae in biopsy or culture samples. PTLD was discarded since the ulcerations were not preceded by a mass-like lesion, the biopsy did not show any characteristics of lymphoproliferative disorder and no systemic symptoms were detected.

Oral ulcers are also associated with immunosuppressive medication due to its direct toxic effect. Vallejo *et al.* found a recurrent oral ulcer in hepatic transplant recipient treated with tacrolimus even within the therapeutic range of dose (8). In our patient, the dose of tacrolimus was constantly monitored and adjusted in order to maintain the therapeutic immunosuppressive effect while minimizing its toxic effect. Therefore, we cannot correlate the appearance of ulceration with high or toxic tacrolimus doses, since these lesions developed when the drug was in the lowest blood concentration for at least two months.

Plana-Pla *et al.* have described the association of a painful oral ulcer with mycophenolate mofetil (MMF) treatment that remitted with suspension of immunosuppressive drugs (9). Contrarily, the temporary suspension of MMF due to our patient’s leukopenia failed to show improvement of oral lesions and the restoring of this drug did not show the appearance of new ulcers.

Parasitic infection in the oral cavity has not been well studied and it is thought to be underreported. There are only three species of *Trichomonas* that can colonize human tissues: *T. hominis*, *T. tenax* and *T. vaginalis*. Only the latter one can cause disease being one of its very rare manifestations the appearance of painful ulcers on the genitals even in immunocompetent patients(10). None but T. tenax is an habitual commensal of oral cavity transmitted by direct contact and is considered to be non-pathogenic but it has been associated to periodontal disease (11). In addition, *T. tenax* is capable of disorganizing three-dimensional spheroid structures of fibroblasts and epithelial gingival cells after 6 hours of interaction. Moreover, it has been demonstrated cytotoxic effects in mammalian cell cultures, similar to those shown by *T. vaginalis* (5).

Humans can accidentally host flagellates that infect other animal species. Parasites such as *Trichomonas gallinae* have been found in the upper airways of humans and phylogenetic studies have shown a close relationship between *T. gallinae* and *T. tenax* (12).

Although it is not possible for us neither to identify the strain of *Trichomonas* nor to prove causality between the presence of the protozoan and the development of oral ulceration in our patient, the clinical response to treatment and the absence of other recognized pathogens indicate that this parasite was likely acting as a pathogen. In conclusion, and to the best of our knowledge, this is the first case in literature that highlights the possibility that *Trichomonas spp.* should be considered as a potential etiological factor in pathogenesis of oral disorders in immunosuppressed patients.

## References

[1] Osiak M, Szubińska-Lelonkiewicz D, Wychowański P, Karakulska-Prystupiuk E, Jędrzejczak W, Wojtowicz A (2018). Frequency of Pathologic Changes in the Oral Cavity in Patients Subjected to Long-term Pharmacologic Immunosuppressive Therapy After Kidney, Liver, and Hematopoietic Cell Transplantation. Transplant Proc.

[2] Marty M, Lemaitre M, Kémoun P, Morrier JJ, Monsarrat P (2017). Trichomonas tenax and periodontal diseases: A concise review. Parasitology.

[3] Kucknoor AS, Mundodi V, Alderete JF (2009). Genetic identity and differential gene expression between Trichomonas vaginalis and Trichomonas tenax. BMC Microbiol.

[4] Yamamoto A, Asaga E, Nagao E, Igarashi T, Goto N (2000). Characterization of the cathepsin B-like proteinases of Trichomonas tenax ATCC 30207. Oral Microbiol Immunol.

[5] Ribeiro LC, Santos C, Benchimol M (2015). Is Trichomonas tenax a Parasite or a commensal?. Protist.

[6] Kaisar MO, Kirwan RM, Strutton GM, Hawley CM, Mudge DW, Campbell SB (2008). Cutaneous manifestations of cytomegalovirus disease in renal transplant recipients: A case series. Transpl Infect Dis.

[7] Terai H, Ueno T, Suwa Y, Omori M, Yamamoto K, Kasuya S (2018). Candida is a protractive factor of chronic oral ulcers among usual outpatients. Jpn Dent Sci Rev.

[8] Vallejo Hernández G, Jiménez C, Arriba L, Moreno E, Lucas M (2001). Resolution of oral ulcerations after decreasing the dosage of tacrolimus in a liver transplantation recipient. Oral Surg Oral Med Oral Pathol Oral Radiol Endod.

[9] Plana-Pla A, Solé LC, Garcia AB, Valdemoros RL (2019). Mycophenolate mofetil-induced mouth ulcers in a kidney transplant patient: Case report and literature review. Nefrología.

[10] Mitchell L, Hussey J (2010). Trichomonas vaginalis: An unusual presentation. Int J STD AIDS.

[11] Benabdelkader S, Andreani J, Gillet A, Terrer E, Pignoly M, Chaudet H (2019). Specific clones of Trichomonas tenax are associated with periodontitis. PLoS One.

[12] Quillfeldt P, Schumm YR, Marek C, Mader V, Fischer D, Marx M (2018). Prevalence and genotyping of Trichomonas infections in wild birds in central Germany. PLoS One.

